# A202 INVESTIGATION OF THE DIRECT EFFECT OF HEMIN-HEMOPEXIN AND HAPTOGLOBIN STIMULATION IN INFLAMMATORY BOWEL DISEASE

**DOI:** 10.1093/jcag/gwac036.202

**Published:** 2023-03-07

**Authors:** A S Ajayi, R Hajjar, M Oliero, T Cuisiniere, C Gerkins, G Fragoso, A Calve, H Vennin-Rendos, M M Santos

**Affiliations:** 1 Nutrition and Microbiome Laboratory, Institut du cancer de Montreal, Centre de recherche du Centre hospitalier de l’Université de Montréal (CRCHUM); 2 Department of Surgery, Faculty of Medicine, Université de Montreal; 3 Department of Medicine, Faculty of Medicine, Université de Montréal, Montreal, QC, Canada

## Abstract

**Background:**

Inflammatory bowel disease (IBD) is a global health issue defined by persistent intestinal epithelial inflammation resulting in bleeding, and it is one of the major risk factors of colorectal cancer (CRC). Hemin is an activator of heme-oxygenase and has anti-inflammatory, cytoprotective, and antiapoptotic properties by stimulating hemopexin and haptoglobin production. Hemopexin and haptoglobin are scavenging proteins with high affinity for hemin and hemoglobin, and are activated by interleukin (IL)-22.

**Purpose:**

To evaluate the protective role of hemin through the stimulation of hemopexin and haptoglobin in experimental acute colitis.

**Method:**

Mice were preconditioned with hemin by intraperitoneal injection with 5mg/kg body weight of hemin for three days while control mice were treated with PBS. Colitis was induced by giving 2.5-3% dextran sulfate sodium (DSS) in drinking water for 12 days. The disease activity index (DAI) was determined by daily monitoring of body weight, fecal bleeding and stool consistency. At the end of the experiment, liver, feces, serum and colon samples were harvested for the evaluation of fecal levels of hemopexin, fecal haptoglobin, IL-22, IL-22BP, IL-6 and TNF-α. Fecal microbiota composition will be determined using 16S rRNA sequencing.

**Result(s):**

We found that hemin treatment resulted in the presence of hemopexin and haptoglobin in fecal samples, where they may exert a protective role and influence the growth of pathobionts able to acquire iron using heme and hemoglobin as sources.

**Conclusion(s):**

The findings from this study will contribute to the understanding of the possible protective role of hemopexin and haptoglobin in ulcerative colitis.

**Please acknowledge all funding agencies by checking the applicable boxes below:**

CIHR

**Disclosure of Interest:**

None Declared

